# Postoperative GH and Degree of Reduction in IGF-1 Predicts Postoperative Hormonal Remission in Acromegaly

**DOI:** 10.3389/fendo.2021.743052

**Published:** 2021-11-18

**Authors:** Tyler Cardinal, Casey Collet, Michelle Wedemeyer, Peter A. Singer, Martin Weiss, Gabriel Zada, John D. Carmichael

**Affiliations:** ^1^ University of Southern California (USC) Pituitary Center, Department of Neurosurgery, Keck School of Medicine of University of Southern California, Los Angeles, CA, United States; ^2^ Department of Medicine, Division of Endocrinology and Diabetes, Keck School of Medicine of University of Southern California, Los Angeles, CA, United States

**Keywords:** acromegaly, transsphenoidal resection, growth hormone, IGF- I, hormonal remission

## Abstract

**Purpose:**

Determine predictive factors for long-term remission of acromegaly after transsphenoidal resection of growth hormone (GH)-secreting pituitary adenomas.

**Methods:**

We identified 94 patients who had undergone transsphenoidal resection of GH-secreting pituitary adenomas for treatment of acromegaly at the USC Pituitary Center from 1999-2019 to determine the predictive value of postoperative endocrine lab values.

**Results:**

Patients underwent direct endoscopic endonasal (60%), microscopic transsphenoidal (38%), and extended endoscopic approaches (2%). The cohort was 63% female and 37% male, with average age of 48.9 years. Patients presented with acral enlargement (72, 77%), macroglossia (40, 43%), excessive sweating (39, 42%), prognathism (38, 40%) and frontal bossing (35, 37%). Seventy-five (80%) were macroadenomas and 19 (20%) were microadenomas. Cavernous sinus invasion was present in 45%. Available immunohistochemical data demonstrated GH staining in 88 (94%) and prolactin in 44 (47%). Available postoperative MRI demonstrated gross total resection in 63% of patients and subtotal resection in 37%. Most patients (66%) exhibited hormonal remission at 12 weeks postoperatively. Receiver operating characteristic (ROC) curves demonstrated postoperative day 1 (POD1) GH levels ≥1.55ng/mL predicted failure to remit from surgical resection alone (59% specificity, 75% sensitivity). A second ROC curve showed decrease in corrected insulin-like growth factor-1 (IGF-1) levels of at least 37% prognosticated biochemical control (90% sensitivity, 80% specificity).

**Conclusion:**

POD1 GH and short-term postoperative IGF-1 levels can be used to successfully predict immediate and long-term hormonal remission respectively. A POD1 GH cutoff can identify patients likely to require adjuvant therapy to emphasize clinical follow-up.

## Introduction

Acromegaly is a disease of excess growth hormone (GH) and insulin-like growth factor-1 (IGF-1) levels, with GH-secreting pituitary adenomas causing 98% of cases (1, 2). Patients with acromegaly experience skeletal and soft tissue overgrowth, particularly in the face and hands. Acromegaly is associated with metabolic, cardiac, and musculoskeletal comorbidities which contribute to higher rates of mortality (2–4). Disease remission is typically defined by postoperative normalization of age- and sex-adjusted IGF-1 levels, random GH levels less than 1 μg/liter, or a nadir of less than 0.4 μg/liter during oral glucose tolerance test (OGTT) (5). Treatment is essential as normalization of GH and IGF-1 levels reduces patient mortality and morbidity progression (4, 6, 7).

Surgical resection alone has shown to be effective in controlling disease in up to 80% of patients with microadenomas and 50% of patients with macroadenomas, with long-term disease control occurring in up to 65% of cases (1, 2). In cases of refractory or residual disease, surgery is typically combined with postoperative medical therapy including somatostatin receptor ligands (SRL), GH-receptor antagonists, dopamine agonists (DA), and radiotherapy (1, 2). Adjuvant therapy is initiated based on remission status as assessed at 3 month postoperative follow-up, and investigating remission predictors is imperative in facilitating decisions regarding postoperative management (3). Current literature suggests various predictors including patient sex, preoperative pituitary deficit, tumor size, invasion characteristics, as well as endocrine lab values (3, 6, 8–12). More specifically, some studies have suggested postoperative GH levels and decreases in IGF-1 from pre- to postoperative levels as predictors of long-term remission (11, 13–15). In a prior study, we identified preoperative IGF-1 and postoperative day 1 (POD1) GH levels as predictors of postoperative remission in a series restricted to endoscopic approaches; however our statistical power was limited by sample size (16). In this study we analyze a series of 94 patients with acromegaly who underwent surgical resection of pituitary adenomas between June 1999 and December 2019 in order to better understand the predictive value of POD1 GH and IGF-1 levels.

## Methods

### Patient Cohort and Evaluation

We retrospectively reviewed our USC Pituitary Center RedCap database of operations performed by two neurosurgeons (GZ and MW) at Los Angeles County + University of Southern California (LAC + USC) Medical Center and Keck Hospital of USC between June 1999 and December 2019. We identified 101 patients with acromegaly who underwent either microscopic or endoscopic endonasal transsphenoidal resection of GH-secreting pituitary adenomas. Seven patients without follow-up IGF-1 levels were excluded, leaving 94 patients included for final analysis. For each surgery, the pathology, type of exposure, presence of intraoperative CSF leak and complications were recorded. Based on Schroeder et al.’s conclusion that a small percentage of tumors from clinically acromegalic patients do not stain positive for GH on immunohistochemistry (IHC), one patient without a pathologically confirmed GH-secreting tumor but with significantly elevated preoperative IGF-1 levels and acromegalic signs and symptoms was included (17). The institutional review board at Keck School of Medicine approved the study (HS-11-00702).

### Neuro-Imaging Evaluation

All patients underwent preoperative MRI to assess tumor size, presence of cavernous sinus invasion, infrasellar invasion, suprasellar extension, sphenoid anatomy, carotid anatomy, anatomy of normal pituitary gland, and position of the optic chiasm and nerves. Patients underwent MRI at 3 months postoperatively to assess extent of resection (EOR), and then annually to monitor tumor progression or recurrence. EOR was defined as either gross total resection (GTR) if no tumor was visible on postoperative MRI or subtotal resection (STR) in cases of observed residual disease. Tumor size was defined by maximal diameter in either the anteroposterior, lateral, or craniocaudal dimension. Parasellar extension into the cavernous sinus(es) was assessed according to the Knosp classification, consisting of 4 grades based on proximity to the ICA (18). Knosp scores were additionally dichotomized into less than or equal to 2 and greater than 2 for statistical analysis.

### Endocrinological Evaluation

Preoperative IGF-1 levels were obtained in all patients to confirm the diagnosis of acromegaly. GH levels were not routinely obtained preoperatively due to its low clinical utility because of its pulsatile secretion. Patients additionally underwent full preoperative endocrine panels to screen for other endocrinopathies. Cabergoline, bromocriptine, and SRL medications were discontinued when decision for surgery was made, on average 4-6 weeks preoperatively. Postoperative GH levels were obtained on POD1, and follow-up IGF-1 levels were obtained at 6 and 12 weeks postoperatively to assess for remission. Hormonal remission was defined as normalization of age- and sex-normalized IGF-1 levels at 12 weeks postoperatively, though IGF-1 levels obtained prior to this were used as indicators of future remission. OGTT and GH levels were used to assess for remission only in patients with postoperative IGF-1 reduction that did not continue to fall within normal limits or that subsequently rose in the setting of symptom resolution. OGTT was performed after 12 weeks postoperatively. For the purposes of between-subject comparison, when available, IGF-1 levels were corrected using the age- and sex-specific upper limit of normal (ULN) and expressed as a percentage of ULN (IGF-1 level/IGF-1 ULN*100). Biochemical control was defined as normalization of IGF-1 with the use of adjuvant therapy in the postoperative period. GH and IGF-1 assays were all sent to Quest Diagnostics laboratories, where assay techniques have changed during the study period according to Quest’s practices. GH assays were performed *via* the RIA technique. Postoperative assessment of pituitary function was performed to assess for hormone deficit recovery or new hypopituitarism.

### Surgical Technique

Patients underwent either microscopic endonasal transsphenoidal, endoscopic endonasal (direct) transsphenoidal, or rarely endoscopic endonasal extended approaches for tumor resection.

### Statistical Evaluation

Continuous variables were assessed for normality of distribution using the Kolmogorov-Smirnov test and are displayed as the means and standard deviation (SD), or medians with interquartile ranges (IQR). Categorical variables are displayed as frequencies. Pearson’s chi-squared analysis was used to compare categorical variables. Correlations were determined using Spearman’s rank-order correlation. Means were assessed using independent samples t-test or Mann-Whitney U test based on distribution. Medians were compared using nonparametric statistical analyses. Multivariate regression was performed to analyze the utility of POD1 GH and IGF-1 as independent predictors of remission to account for both preoperative maximal tumor diameter and postoperative extent of resection (EOR). Receiver operator curves (ROC) were generated to determine predictive cut-off points. Statistical analysis was performed using SPSS 25.0 statistical software (IBM Corp., Armonk, NY) and a 2-tailed *p*-value <0.05 was considered significant.

## Results

### Patient Demographics and Presentation

Of the 94 patients included in this study, 59 (63%) were female and 35 (37%) were male. Mean age at surgery was 48.9 years (SD 13.7). Seventeen patients (18%) underwent surgery at LAC + USC Medical Center and the other 77 (82%) were operated on at Keck Hospital. Thirty-eight patients (40%) presented with headache, 22 (23%) with fatigue, 15 (16%) with visual changes, 11 (12%) with decreased libido, 13 (14%) with amenorrhea/oligomenorrhea, and 5 (5%) with galactorrhea. Four patients (4%) had incidentally discovered pituitary adenomas with subsequent diagnosis of acromegaly. All patients except one presented with typical signs and symptoms of acromegaly including acral enlargement in 72 patients (77%) and facial changes in 60 patients (64%). The most common facial changes included macroglossia (40 patients, 43%), prognathism (38 patients, 40%), frontal bossing (35 patients, 37%), and teeth gaps (27 patients, 29%). Thirty-nine patients (42%) reported excessive sweating, 31 (33%) had skin tags, 24 (26%) reported snoring, 22 (23%) had oily/thickened skin, and 18 (19%) had arthralgias. The patient who did not present with typical signs or symptoms of acromegaly was 13 years old when the pituitary adenoma was discovered on CT scan as part of sinusitis workup, but he had always been above the 90^th^ percentile for height.

On preoperative neurological exam, 5 patients (5%) had visual acuity loss, 5 (5%) had visual field cuts, and 1 (1%) had cranial nerve IV palsy. The majority (83 patients, 88%) had no focal neurological deficits. Twelve patients (13%) were treated with medications preoperatively for disease control, including bromocriptine in 2 (2%), cabergoline in 4 (4%), and somatostatin receptor ligands (SRL) in 7 (7%) (**Table 1**).

**Table 1:** Presenting patient characteristics, prior treatment, and endocrine status and lab values.

Patient characteristics	No. (%)
Age at surgery (years)	48.9 ± 13.7
Male:female ratio	1:1.69
Keck Hospital	77 (82)
LAC + USC Medical Center	17 (18)
**Prior treatment**	**No. (%)**
Transsphenoidal resection	9 (10)
Medications	12 (13)
Craniotomy	2 (2)
Radiosurgery	2 (2)
Radiation therapy	1 (1)
None	76 (81)
**Preoperative endocrine status**	**No. (%)**
Acromegaly	94 (100)
Hyperprolactinemia	15 (16)
Low gonadotroph axis	12 (13)
Hypothyroidism	9 (10)
Low cortisol axis	2 (2)
Panhypopituitarism	1 (1)
**Preoperative endocrine labs**	**Median (IQR)**
IGF-1 (ng/ml) (n=82)	714.5 (510-877)
Corrected IGF-1 (%ULN) (n=63)	238 (165-302)
Growth hormone (ng/ml) (n=51)	13.4 (5.6-36)

### Preoperative Endocrine Status

All patients met clinical criteria for acromegaly and presented with elevated IGF-1 levels. Other preoperative endocrinopathies are presented in **Table 1**. Hyperprolactinemia was suspected to be secondary to prolactin cosecretion in 3 of the 15 presenting with hyperprolactinemia (20%) and stalk effect in the other 12 (80%). Preoperative GH levels are not routinely obtained due to their pulsatile nature but were available for 51 patients (54%) with a median value of 13.4ng/mL (IQR 5.6-36).

Corrected preoperative IGF-1 levels were available for 63 of the 94 included patients (67%), with a median value of 238ng/mL (IQR 165-302). Elevated preoperative IGF-1 levels were available for 82 patients (87%) with a median value of 714ng/mL (IQR 510-877). The other 12 patients (13%) had reports of elevated preoperative IGF-1 levels that were unable to be accessed as they were performed at outside laboratories. For the 82 patients with preoperative IGF-1 levels available, there was a significant correlation between preoperative IGF-1 levels and tumor size (p=0.001) (**Table 1**).

### Preoperative Neuro-Imaging Characteristics

Preoperative tumor size was available for 77 patients (82%) with a mean maximal diameter of 1.8 cm (SD 1). The majority were macroadenomas (75, 80%) and 19 (20%) were microadenomas. Five (5%) were giant pituitary adenomas (>4cm). Suprasellar invasion was observed in 27 patients (29%), cavernous sinus invasion in 42 patients (45%), and infrasellar extension in 28 patients (30%). Knosp scoring was available for 60 patients (64%) with a score of 0 in 21 (22%), a score of 1 in 21 (22%), a score of 2 in 9 (10%), a score of 3 in 6 (6%) and a score of 4 in 3 (3%). Purely intrasellar tumors were found in 24 patients (26%) (**Table 2**).

**Table 2:** Preoperative neuroimaging, pathology, and immunohistochemical staining.

Preoperative neuroimaging	No. (%)
Maximal tumor diameter (cm)	1.8 ± 1.0
Suprasellar extension	27 (29)
Infrasellar invasion	28 (30)
Cavernous sinus invasion	42 (45)
No invasion	24 (26)
Macroadenoma	75 (80)
Microadenoma	19 (20)
Knosp score	
*0*	21 (22)
*1*	21 (22)
*2*	9 (10)
*3*	3 (6)
*4*	4 (3)
**Pathology**	**No. (%)**
Somatotroph	83 (88)
Mammosomatotroph	9 (10)
Nonfunctional adenoma	1 (1)
**Immunohistochemical staining**	**No. (%)**
Growth hormone	88 (94)
Prolactin	44 (47)
Thyroid-stimulating hormone	19 (20)
Luteinizing hormone	6 (6)
Follicle-stimulating hormone	6 (6)
Adrenocorticotropin hormone	5 (5)

### Surgery Details and Intraoperative CSF Leaks

The majority of patients (56, 60%) underwent EETS, 36 patients (38%) underwent microscopic transsphenoidal surgery, and 2 (2%) underwent an extended endoscopic approach. Intraoperative CSF leak occurred in 28 cases (30%) and were repaired with fat (25, 27%), fascia (21, 22%), dural substitute (3, 3%), dural sealant (2, 2%), pedicled naso-septal flap (2, 2%), and lumbar drain (1, 1%). No significant difference was found between POD1 GH levels (*p* = 0.137) or hormonal remission outcomes (*p*=0.257) and surgical technique.

### Pituitary Adenoma Pathology and Immunostaining

Most patients were diagnosed with purely GH-secreting pituitary adenomas based on histopathology (83, 88%). Nine patients (10%) were diagnosed with mammosomatotroph adenomas, one of whom did not present with hyperprolactinemia, and 1 patient (1%) was diagnosed with a non-functional adenoma based on immunohistochemistry but was considered to have acromegaly based on preoperatively elevated IGF-1 that decreased postoperatively, combined with acromegalic features. In 1 patient (1%) pathology was not available, but elevated preoperative IGF-1 levels consistent with acromegaly decreased postoperatively to within normal limits. In patients with available immunohistochemical (IHC) staining, 88 (94%) stained positive for GH, 44 (47%) also stained positive for prolactin, 19 (20%) also stained positive for TSH, 6 (6%) also stained positive for LH, 6 (6%) also stained positive for FSH, 5 (5%) also stained positive for ACTH, and 1 (1%) also was the null-cell adenoma referred to above. CAM5.2 staining was performed in 25 tumors, with positive staining in 24 (96%).

### Postoperative Complications, Readmissions, and Reoperations

The mean hospital stay was 2.6 days (SD 2). Within the 30-day postoperative period, 12 patients (13%) had hyponatremia, 6 (6%) had epistaxis, 5 (5%) experienced postoperative CSF leaks, 3 (3%) had transient diabetes insipidus (DI), 2 (2%) became septic, 1 (1%) had permanent DI, 1 (1%) experienced meningitis, and 1 (1%) experienced sinusitis. No patients included in this series died.

Early readmission within a 30-day postoperative period occurred in 7 patients (7%), all for hyponatremia. One patient (1%) underwent early reoperation for CSF leak repair. CSF leak self-resolved without intervention in the other 4 patients.

### Surgical Outcomes

Postoperative MRI was available for 67 patients (71%) and demonstrated gross total resection (GTR) in 42 (63%) and subtotal resection (STR) in 25 (37%). Median imaging follow-up time was 16 months (IQR 4.8-38). Disease recurrence after GTR occurred in 3 patients (3%) with a mean time of 16 months (SD 14). Progression of residual disease occurred in 2 patients (2%) at a mean time of 20 months (SD 15). EOR was significantly correlated with POD1 GH levels, with lower levels being observed in patients who underwent GTR (U=223, *p*<0.001). Furthermore, patients who underwent GTR were significantly more likely to achieve postoperative remission (*p*=0.02).

### Endocrinological Outcomes

The median POD1 GH level was 1.7ng/mL (IQR 0.9-3.3). All patients were followed in clinic postoperatively with a median clinical follow-up time of 17 months (IQR 4.6-41). The majority (62 patients, 66%) experienced hormonal remission at a median time of 3 months (IQR 1-5). Twenty-four patients (26%) received additional treatment, 15 of which (16%) ended up achieving biochemical control. Eight patients (9%) did not receive additional treatment and did not achieve hormonal remission before being lost to follow-up at a mean of 3 months (SD 2). Follow-up IGF-1 levels were available for all patients except 1 in which there was no follow-up IGF-1 but GH suppression was observed during OGTT. At most recent clinical follow-up, the median IGF-1 level was 190ng/mL (IQR 138.5-274) and apex level (as defined by highest recorded postoperative IGF-1) was 238ng/mL (IQR 175-370.5). Corrected IGF-1 scores were available for 71 patients (76%) with a median of 62.8 (IQR 45-80) for most recent levels and 80.4 (IQR 64-114) for apex levels.

**Figure 1:**
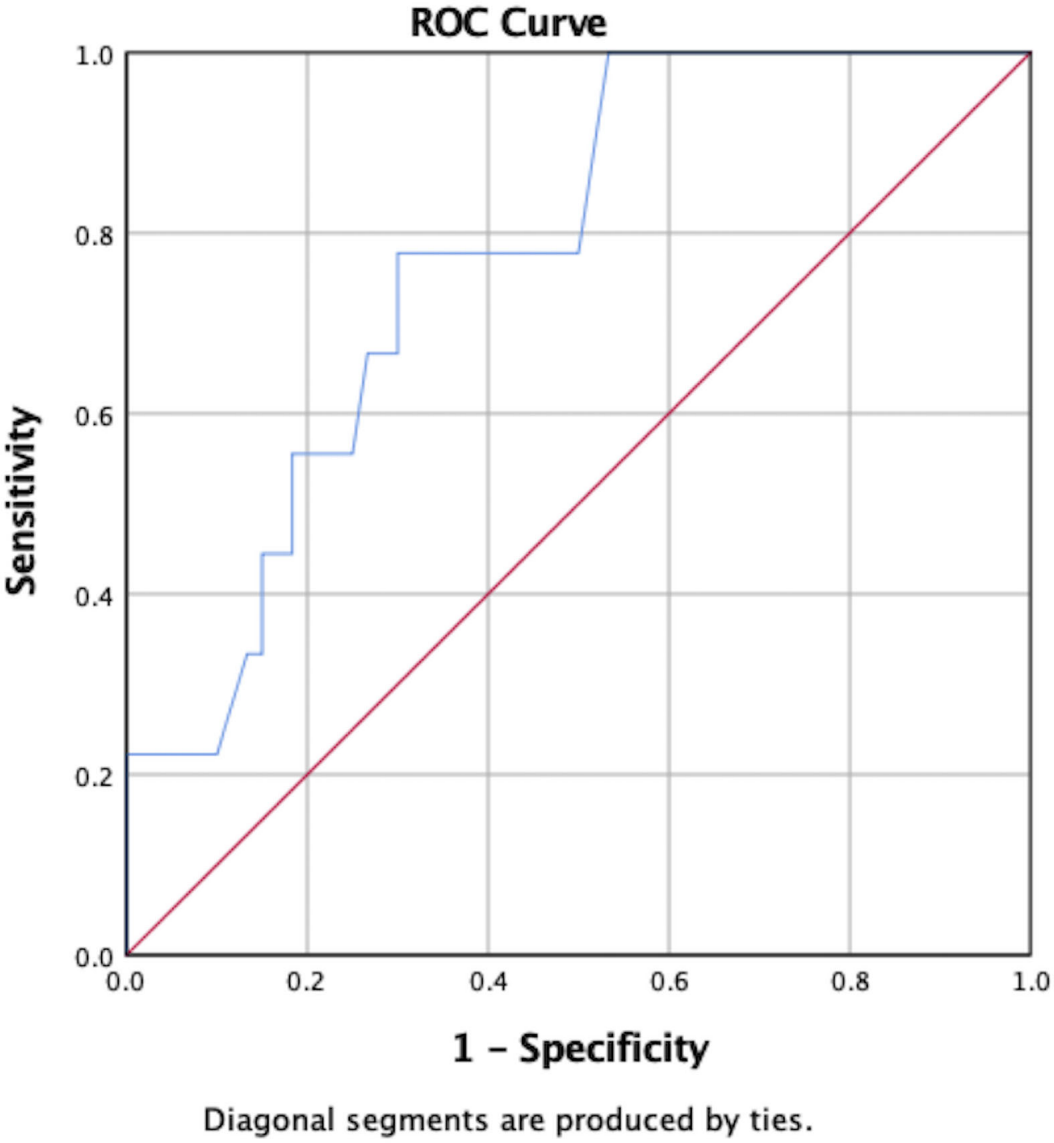
ROC curve demonstrating predictive ability of POD1 GH to predict non-remission.

POD1 GH levels were significantly associated with hormonal remission (U=105, *p*=0.011). Specifically, the mean POD1 GH level in patients who achieved hormonal remission was 1.60ng/mL *versus* 9.51ng/mL in patients who did not remit. A ROC curve predicted hormonal non-remission with a sensitivity of 75% and a specificity of 59% in patients whose POD1 GH levels were greater than 1.55ng/mL (AUC=0.78, 95%CI 0.63–0.93) (**Figure 1**). POD1 GH remained an independent predictor of remission on multivariate analysis including EOR and preoperative maximal tumor diameter (*p*=0.001).

**Figure 2:**
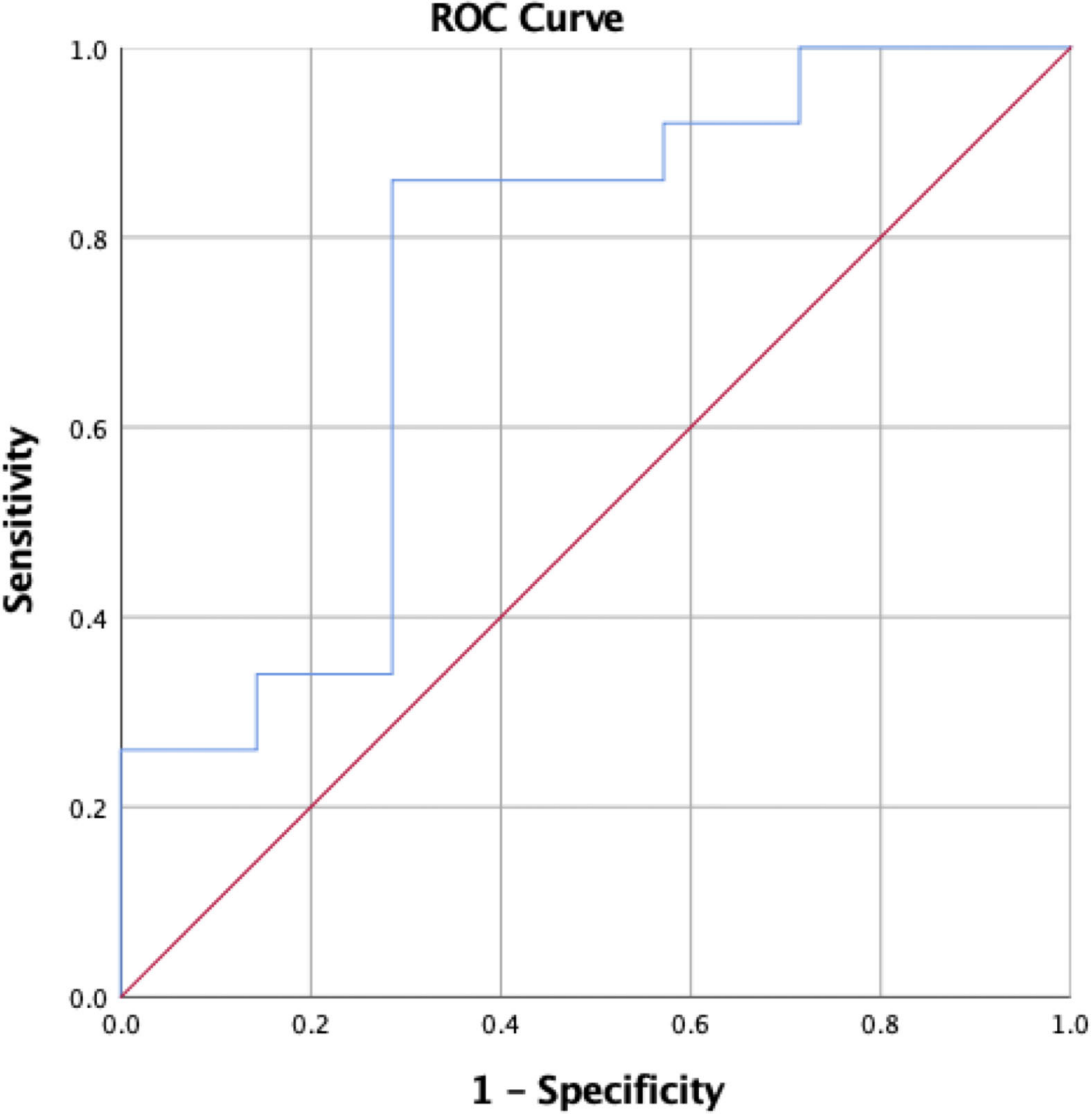
ROC curve demonstrating change in IGF-1 from preoperative to 6-12 week postoperative levels as a predictor of postoperative hormonal remission.

A ROC curve demonstrated that a 37% or greater decrease in IGF-1 from preoperative levels as measured at 6-12 weeks postoperatively prognosticated hormonal remission with a sensitivity of 90% and specificity of 80% (AUC=0.82, 95%CI 0.55–1.0) (**Figure 2**). In some patients (n=49), the exact week at which postoperative IGF-1 levels were measured was unavailable. These patients were excluded from this predictive analysis, which included the other 45 patients with corrected IGF-1 levels measured between 6 and 12 weeks postoperatively. On multivariate analysis, IGF-1 levels were not a significant predictor of remission when EOR was taken into account, though preoperative tumor size did not have a significant impact (*p*=0.575). All patients on preoperative SRL medications were excluded from POD1 GH analyses due to their potential influence on GH levels. Pre-treatment IGF-1 levels were used for analysis of delta IGF-1 levels.

### Additional Treatment

Of the 24 patients who underwent additional treatment, 8 (9%) underwent adjuvant stereotactic radiosurgery, 2 (2%) received external radiation therapy, and 1 (1%) underwent additional surgical resection. Twenty-one patients (22%) received adjuvant medical therapy with bromocriptine (1, 1%), cabergoline (9, 10%), SRL (15, 16%), and pegvisomant (1, 1%). Of patients who received additional treatment, 15 (16%) exhibited biochemical control after treatment and 9 (10%) had refractory disease. Seven of those who did not achieve biochemical control (7%) were lost to follow-up. The other 2 (2%) have known residual disease that has been deemed unresectable and continue to be in non-remission despite treatment with SRL in both and adjuvant radiotherapy in one.

In order to predict the need for additional treatment in this patient population, an additional analysis was performed after eliminating the 9 previously mentioned patients who did not undergo hormonal remission but were lost to follow-up without initiation of adjuvant therapy. Mean POD1 GH levels were 5.9ng/mL (SD 8) in patients who received additional treatment, compared to 1.5ng/mL in patients who did not (U=257, *p*<0.001).

### Comparison of Pre- and Postoperative Characteristics Based on Suggested POD1 GH Cutoff

In order to better understand the value of the proposed POD1 GH cutoff of 1.55ng/mL and its relationship with pre- and postoperative characteristics, patients were divided into two groups according to POD1 GH level (**Tables 3** and **4**). Patients with POD1 GH less than 1.55ng/mL were more likely to have lower median preoperative GH levels (6.8ng/mL *vs*. 17.2ng/mL, *p*=0.025), and smaller mean maximal tumor diameter (1.5cm *vs*. 2.1cm, *p*=0.012). However, no significant differences were noted in patient demographics, prior treatment, preoperative IGF-1 levels, or tumor invasion characteristics. Patients who underwent GTR were more likely to have POD1 GH levels less than 1.55ng/mL (54.5% *vs*. 36%, *p*=0.003). Patients with POD1 GH levels less than 1.55ng/mL were additionally more likely to undergo hormonal remission (91% *vs*. 72%, *p*=0.02), and less likely to receive additional treatment, specifically adjuvant medical therapy (4.5% *vs*. 38%, *p*<0.001).

**Table 3:** Comparison of presenting patient characteristics, prior treatment, endocrine lab values, and preoperative neuroimaging based on POD1 GH cutoff of 1.55ng/mL.

	POD1 GH <1.55ng/mL (n = 44)	POD1 GH ≥1.55ng/mL (n = 50)	p-value
**Patient characteristics**			
Age at surgery (years)	48.4 ± 10.9	49.4 ± 15.9	0.749
Male:female ratio	1:1.45	1:1.94	0.489
**Prior treatment**	**No. (%)**	**No. (%)**	
Transsphenoidal resection	2 (4.5)	7 (14)	0.120
Medications	5 (11)	7 (14)	0.702
None	37 (84)	38 (76)	0.330
**Preoperative endocrine labs**	**Median (IQR)**	**Median (IQR)**	
IGF-1 (ng/ml) (n=82)	714 (507-886)	715 (511-874)	1.00
Corrected IGF-1 (%ULN) (n=63)	239 (165-300)	232 (154-306)	1.00
Growth hormone (ng/ml) (n=51)	6.8 (3.4-19.5)	17.2 (10.6-51.3)	0.025
**Preoperative neuroimaging**	**No. (%)**	**No. (%)**	
Maximal tumor diameter (cm)	1.5 ± 0.7	2.1 ± 1.2	0.012
Macroadenoma	33 (75)	42 (84)	0.278
Microadenoma	11 (25)	8 (16)	0.278
Suprasellar extension	9 (21)	18 (36)	0.096
Infrasellar invasion	14 (32)	14 (28)	0.686
Cavernous sinus invasion	18 (41)	24 (48)	0.490
No invasion	14 (32)	10 (20)	0.190
Knosp score			
*Less than 2*	29 (66)	22 (44)	0.055
*3 or 4*	2 (4.5)	14 (24)	0.055

**Table 4:** Overall surgical and clinical outcomes and comparisons based on POD1 GH cutoff of 1.55ng/mL.

	Overall (n = 94)	POD1 GH <1.55ng/mL (n = 44)	POD1 GH ≥1.55ng/mL (n = 50)	p-value
**Surgical outcomes**	**No. (%)**	**No. (%)**	**No. (%)**	
Median imaging follow-up (months)	16 (4.8-38)	18 (IQR 8.8-40)	15 (IQR 3.4-37)	–
Gross total resection	42 (63)	24 (54.5)	18 (36)	0.003
Subtotal resection	25 (37)	5 (11)	20 (40)	0.003
Recurrence after GTR	3 (3)	2 (4.5)	1 (2)	0.555
Progression after STR	2 (2)	1 (2)	1 (2)	0.824
**Clinical outcomes**	**No. %)**	**No. (%)**	**No. (%)**	
Median clinical follow-up (months)	17 (4.6-41)	17 (IQR 5.5-44)	16 (IQR 3.4-38)	–
Hormonal remission	77 (82)	40 (91)	37 (74)	0.034
Time to remission (months)	8 (IQR 1-8.5)	3 (IQR 1-5)	4 (IQR 1.5-10.5)	–
Additional treatment	24 (26)	4 (9)	30 (60)	0.001
Medications	21 (22)	2 (4.5)	19 (38)	<0.001
Stereotactic radiosurgery	8 (9)	3 (7)	5 (10)	0.581
Radiation therapy	2 (2)	1 (2)	1 (2)	0.927
Additional surgical resection	1 (1)	1 (2)	0 (0)	0.284
**Postoperative endocrine labs**	**Median (IQR)**	**Median (IQR)**	**Median (IQR)**	
Most recent follow-up IGF-1 (ng/ml) (n=93)	190 (138.5-274)	194 (139-235)	187 (137-340)	0.836
Most recent follow-up corrected IGF-1 (%ULN) (n=71)	62.8 (45-80)	60.7 (43.8-78.7)	63 (46.6-91.9)	1.00
Apex follow-up IGF-1 (ng/ml) (n=93)	238 (175-370.5)	226 (165-316)	260 (182-433)	0.097
Apex follow-up corrected IGF-1 (%ULN) (n=71)	80.4 (64-114)	74.2 (61.6-97.9)	91.8 (64.1-162)	0.157

## Discussion

In this study, we analyzed patient data from 94 surgical cases with long term follow-up treated at a tertiary pituitary center in order to investigate the utility of endocrine lab values in predicting postoperative hormonal remission in acromegaly. All patients underwent pituitary adenoma resection, either by microscopic, endoscopic, or extended endoscopic transsphenoidal surgery. The two main predictors assessed in this study were POD1 GH levels and degree of change in IGF-1 levels. GH half-life of 20-30 minutes suggests that GH levels within 24 hours postoperatively may reliably predict the success of surgical resection (13). Indeed, a growing body of evidence accurately demonstrating a correlation between postoperative GH levels and remission is emerging (13–17, 19). Previous studies analyzing POD1 GH levels to predict remission proposed optimal thresholds ranging from less than 1.03 to as high as 3.66ng/mL (notwithstanding the fact that assays have changed over time) (11, 13–15). Our findings were concordant with the literature and showed lower POD1 GH levels in patients who achieved postoperative hormonal remission than those who did not remit. We were unable to identify a POD1 GH cutoff that would predict hormonal remission in our patient population with adequate sensitivity and specificity to be used alone. However, we did find that a POD1 GH greater than or equal to 1.55ng/ml predicted hormonal non-remission with a sensitivity of 75% and a specificity of 59%.

In addition to POD1 GH levels, we found a significant correlation between achieving biochemical control and percent decrease in corrected IGF-1 from preoperative to apex postoperative levels. Specifically, a 37% reduction in corrected IGF-1 levels following transsphenoidal resection at 6-12 weeks postoperatively was predictive of immediate hormonal remission or long-term biochemical control with the use of adjuvant therapy with a sensitivity of 90% and a specificity of 80%. Hazer et al. found an uncorrected IGF-1 reduction value of 51.1% at 1 month following transsphenoidal resection predicted remission with a sensitivity of 74.4% and a specificity of 73.7%. Besides the updated use of corrected IGF-1 levels, an important distinction between our findings and Hazer et al.’s is the timing of the measurement of postoperative IGF-1 levels (1 month *vs*. between 6-12 weeks). While IGF-1 levels have been found to fluctuate up to 3 months following resection (20, 21), further studies are merited to explore the optimal window for using IGF-1 reduction as a predictor of surgical outcome. Additionally, the use of corrected IGF-1 values using the upper limit of normal enhances our ability to compare values between individuals and across age-related changes in the normal range (22). Z-scores, which serve a similar purpose to IGF-I correction, were not widely available during the early years of this study and were not able to be analyzed. One limitation, in addition to the retrospective nature of this study, is that scheduling regular postoperative clinical follow-up is particularly difficult at our institution due to our significant percentage of patients in a county healthcare system, and a wide geographical area for tertiary referrals at the Keck Medical Center. Importantly, postoperative IGF-1 level at 6-12 weeks was not a significant independent predictor of remission when EOR was considered on multivariate regression. However, EOR in our study was measured as either STR or GTR and it is unsurprising that GTR is highly influential on postoperative remission. In cases of STR, a postoperative decrease in IGF-1 remains useful in predicting remission.

In the current series, 18% of patients were from the county healthcare system and the other 82% were operated on at our private institution. The patient population were slightly older compared to other acromegaly studies with a mean age of 48.9 ± 13.7 years, and included more females than other studies, with a male/female ratio of 1:1.69. We had a comparable number of patients to other large acromegaly studies, with a similar mean tumor size and distribution of micro- and macroadenomas (3, 10). Mean clinical follow-up time was 30 months and our remission rate was comparable to that found in current acromegaly literature, with 65% remitting postoperatively (3, 6, 8, 9, 11, 16).

Currently it is standard practice to delay initiation of adjuvant therapy for 3-months in order to accurately assess long-term remission status following resection (3). While adjuvant therapies such as radiotherapy requires postoperative MRI to exclude residual tumor shrinkage (13), postoperative biochemical markers may offer guidelines regarding postoperative monitoring and pharmacological treatment. In this study we found POD1 GH levels were lower in patients who achieved disease control with surgery alone than in patients who received adjuvant therapy. Comparable to our POD1 GH cutoff of ≥1.55ng/mL, Cunha et al. found a postoperative random GH level of ≥1.7 ng/ml as a reliable indicator of surgical failure in their patients, though the timeline of GH sampling was unclear (20). Other studies have suggested POD1 GH cutoffs of 2.5ng/mL (23) and 2.33ng/mL (14) to predict postoperative remission, though these higher values reduced the sensitivity while raising the specificity of the cutoff. At our institution, we find increased utility in a cutoff with higher sensitivity to identify patients who may be more likely to require additional treatment.

Early identification of patients requiring adjuvant therapy to achieve biochemical control may help in the planning and execution of postoperative evaluation and medical care. To reduce the number of patients lost to follow up, early identification of patients requiring additional care would also serve to educate patients and the care team, emphasizing the need for further evaluation and treatment in a timely manner. For instance, in our study we found ¾ of patients who were lost to follow-up while not in remission and were candidates for adjuvant therapy had POD1 GH levels >1.55ng/mL. Postoperative clinical follow-up requires coordination between the neuroendocrinologist and neurosurgeon offices as well as laboratory testing and imaging scheduling. Identifying patients while they are still in the hospital who are more likely to require postoperative follow-up would assist with postoperative patient expectation management and allow for emphasis on scheduling clinical follow-up. At follow-up office visits, a POD1 GH cutoff should be used in conjunction with 3-month follow-up MRI and IGF-1 levels to determine remission chances and initiation of adjuvant therapy.

We continue to observe a strong association between POD1 GH levels and EOR, with lower POD1 GH levels being found in patients who underwent GTR of their tumor. As we found here and in current literature, acromegaly remission rates are highly dependent on EOR (24). Therefore, we suggest POD1 GH levels used in combination with the EOR may improve postoperative predictions of patient outcomes and can be used to plan a postoperative treatment approach. Determining EOR is limited, however, by availability of postoperative MRI which can be difficult to coordinate, especially in a county patient population. At our institution, the neurosurgeon and endocrinologist work together using a combination of intraoperative assessment of EOR and POD1 GH levels to determine the postoperative treatment plan. Although intraoperative assessment of GTR has been shown to overlook residual tumor in 15% of cases (25), it has nonetheless been identified as a reliable short-term predictive marker of postoperative cure (1).

This study included patients who underwent both microscopic and endoscopic resection and found similar remission rates between the two surgical techniques, which is consistent with current literature (10). We additionally found that POD1 GH levels did not differ based on surgical technique. This supports the utility of POD1 GH levels in predicting remission in patients undergoing surgical resection of their GH-secreting pituitary adenoma regardless of technique.

Recent studies have identified similar characteristics as predictors of hormonal remission in patients with acromegaly. Predictive models took into account patient age (26), cavernous sinus invasion (26–29), preoperative GH levels (26, 29), EOR (28), and POD1 GH (28) in various combinations to anticipate postoperative remission. Our conclusions are primarily in line with the findings from these studies. A recent structured review of the literature identified cavernous sinus invasion and higher preoperative GH levels as most predictive of failure to achieve postoperative remission, with low postoperative GH predictive of long-term remission (30). In our series, patients with lower POD1 GH level were more likely to have lower median preoperative GH, however this is limited by availability of preoperative GH. This merits future investigation, and though preoperative GH is not utilized for diagnosis, it may be useful as a predictive tool.

## Limitations

This study was primarily limited by its retrospective design. This is primarily reflected in availability of endocrine laboratory values, preoperative imaging, and pathology data, particularly in patients whose data is not available in the electronic health record. Additionally, our mean clinical and imaging follow-up times were complicated by multiple factors, including a transition to electronic medical record system with loss of original records. The loss of original paper charts additionally prevented inclusion of preoperative and corrected IGF-1 levels and availability of pre- and postoperative MRI in some patients (22). Z-scores, which serve a similar purpose to IGF-I correction, were not widely available during the early years of this study and were therefore not able to be analyzed. Another limitation is that scheduling postoperative clinical follow-up is particularly difficult at our institution due to our significant percentage of patients in a county healthcare system, which is unfortunately common in a county system, as well as the wide geographical area for tertiary referrals at the Keck Medical Center. Postoperative IGF-1 levels were measured at some point between 6 and 12 weeks postoperatively based on patient availability, which ideally would have occurred at the same postoperative point across all patients. Additional confirmation of remission with OGTT was not available for most patients as it is only used in equivocal cases, though may be a useful predictor of remission. Analysis of indicators for adjuvant therapy and hormonal remission outcomes was somewhat limited by the 9 patients who were lost to follow-up before adjuvant therapy could be initiated. Due to cost and assay availability, immunostaining at LAC + USC Medical Center is only limited to hormones(s) suspected of being secreted.

## Conclusion

This study supports the utility of POD1 GH as well as the percent decrease in IGF-1 levels as reliable predictors of postoperative hormonal remission in acromegaly patients undergoing transsphenoidal resection. Specifically, we suggest that a POD1 GH level greater than 1.55 ng/mL is predictive of surgical failure and a postoperative IGF-1 level reduction of at least 37% at 6 to 12 weeks is promising for long-term biochemical control. We suggest using a POD1 GH cutoff to identify patients who are less likely to undergo surgical remission and to whom the importance of postoperative clinical follow-up should be emphasized. We recommend that a combination of POD1 GH level, EOR as determined by 3-month postoperative MRI, and the postoperative reduction in IGF-1 level be used conjunctively to predict the need for adjuvant therapy and long-term surgical success. Further research is indicated to explore the ideal period at which postoperative IGF-1 should be measured to best predict remission using the percent reduction from preoperative levels.

## Data Availability Statement

The raw data supporting the conclusions of this article will be made available by the authors, without undue reservation.

## Ethics Statement

The studies involving human participants were reviewed and approved by the institutional review board at the Keck School of Medicine (HS-11-00702). Written informed consent for participation was not required for this study in accordance with the national legislation and the institutional requirements.

## Author Contributions

TC: Formal analysis, investigation, data curation, writing – original draft, and writing – revisions. CC: writing – original draft. MiW: formal analysis, writing – revisions. PS: resources and writing – revisions. MaW: resources and writing – revisions. GZ: resources, conceptualization, methodology, resources, supervision, project administration, and writing – revisions. JC: resources, conceptualization, methodology, resources, supervision, project administration, and writing – revisions. All authors contributed to the article and approved the submitted version.

## Conflict of Interest

JC: Recordati: Honoraria, advisory board Chiasma Principal investigator Crinetics Principal investigator.

The remaining authors declare that the research was conducted in the absence of any commercial or financial relationships that could be construed as a potential conflict of interest.

## Publisher’s Note

All claims expressed in this article are solely those of the authors and do not necessarily represent those of their affiliated organizations, or those of the publisher, the editors and the reviewers. Any product that may be evaluated in this article, or claim that may be made by its manufacturer, is not guaranteed or endorsed by the publisher.

## References

[1] Melmed S. Acromegaly. N Engl J Med (1990) 322:966–77. doi: 10.1056/NEJM199004053221405 2179724

[2] Melmed S Bronstein MD Chanson P Klibanski A Casanueva FF Wass JAH. A Consensus Statement on Acromegaly Therapeutic Outcomes. Nat Rev Endocrinol (2018) 14:552–61. doi: 10.1038/s41574-018-0058-5 PMC7136157 30050156

[3] Albarel F Castinetti F Morange I Conte-Devolx B Gaudart J Dufour H. Outcome of Multimodal Therapy in Operated Acromegalic Patients, a Study in 115 Patients. Clin Endocrinol (Oxf) (2013) 78:263–70. doi: 10.1111/j.1365-2265.2012.04492.x 22783815

[4] Holdaway IM Bolland MJ Gamble GD. A Meta-Analysis of the Effect of Lowering Serum Levels of GH and IGF-I on Mortality in Acromegaly. Eur J Endocrinol (2008) 159:89–95. doi: 10.1530/EJE-08-0267 18524797

[5] Giustina A Chanson P Bronstein MD Klibanski A Lamberts S Casanueva FF. A Consensus on Criteria for Cure of Acromegaly. J Clin Endocrinol Metab (2010) 95:3141–8. doi: 10.1210/jc.2009-2670 20410227

[6] Hofstetter CP Mannaa RH Mubita L Anand VK Kennedy JW Dehdashti AR. Endoscopic Endonasal Transsphenoidal Surgery for Growth Hormone-Secreting Pituitary Adenomas. Neurosurg Focus (2010) 29:E6. doi: 10.3171/2010.7.FOCUS10173 20887131

[7] Pivonello R Auriemma RS Grasso LF Pivonello C Simeoli C Patalano R. Complications of Acromegaly: Cardiovascular, Respiratory and Metabolic Comorbidities. Pituitary (2017) 20:46–62. doi: 10.1007/s11102-017-0797-7 28224405

[8] Freda PU Wardlaw SL Post KD. Long-Term Endocrinological Follow-Up Evaluation in 115 Patients Who Underwent Transsphenoidal Surgery for Acromegaly. J Neurosurg (1998) 89:353–8. doi: 10.3171/jns.1998.89.3.0353 9724106

[9] Jane JA Starke RM Elzoghby MA Reames DL Payne SC Thorner MO. Endoscopic Transsphenoidal Surgery for Acromegaly: Remission Using Modern Criteria, Complications, and Predictors of Outcome. J Clin Endocrinol Metab (2011) 96:2732–40. doi: 10.1210/jc.2011-0554 21715544

[10] Sarkar S Rajaratnam S Chacko G Chacko AG. Endocrinological Outcomes Following Endoscopic and Microscopic Transsphenoidal Surgery in 113 Patients With Acromegaly. Clin Neurol Neurosurg (2014) 126:190–5. doi: 10.1016/j.clineuro.2014.09.004 25278017

[11] Starke RM Raper DM Payne SC Vance ML Oldfield EH Jane JA. Endoscopic *vs* Microsurgical Transsphenoidal Surgery for Acromegaly: Outcomes in a Concurrent Series of Patients Using Modern Criteria for Remission. J Clin Endocrinol Metab (2013) 98:3190–8. doi: 10.1210/jc.2013-1036 23737543

[12] Yildirim AE Sahinoglu M Divanlioglu D Alagoz F Gurcay AG Daglioglu E. Endoscopic Endonasal Transsphenoidal Treatment for Acromegaly: 2010 Consensus Criteria for Remission and Predictors of Outcomes. Turk Neurosurg (2014) 24:906–12. doi: 10.5137/1019-5149.JTN.11288-14.1 25448208

[13] Sarkar S Jacob KS Pratheesh R Chacko AG. Transsphenoidal Surgery for Acromegaly: Predicting Remission With Early Postoperative Growth Hormone Assays. Acta Neurochir (Wien) (2014) 156:1379–87; discussion 1387. doi: 10.1007/s00701-014-2098-5 24781680

[14] Hazer DB Işık S Berker D Güler S Gürlek A Yücel T. Treatment of Acromegaly by Endoscopic Transsphenoidal Surgery: Surgical Experience in 214 Cases and Cure Rates According to Current Consensus Criteria. J Neurosurg (2013) 119:1467–77. doi: 10.3171/2013.8.JNS13224 24074496

[15] Dutta P Korbonits M Sachdeva N Gupta P Srinivasan A Devgun JS. Can Immediate Postoperative Random Growth Hormone Levels Predict Long-Term Cure in Patients With Acromegaly? Neurol India (2016) 64:252–8. doi: 10.4103/0028-3886.177622 26954802

[16] Cardinal T Rutkowski MJ Micko A Shiroishi M Jason Liu CS Wrobel B. Impact of Tumor Characteristics and Pre- and Postoperative Hormone Levels on Hormonal Remission Following Endoscopic Transsphenoidal Surgery in Patients With Acromegaly. Neurosurg Focus (2020) 48:E10. doi: 10.3171/2020.3.FOCUS2080 32480366

[17] Schroeder JL Spiotta AM Fleseriu M Prayson RA Hamrahian AH Weil RJ. Absence of Immunostaining for Growth Hormone in a Subset of Patients With Acromegaly. Pituitary (2014) 17:103–8. doi: 10.1007/s11102-013-0474-4 23475513

[18] Knosp E Steiner E Kitz K Matula C. Pituitary Adenomas With Invasion of the Cavernous Sinus Space: A Magnetic Resonance Imaging Classification Compared With Surgical Findings. Neurosurgery (1993) 33:610–7; discussion 617–8. doi: 10.1227/00006123-199310000-00008 8232800

[19] Kim EH Oh MC Lee EJ Kim SH. Predicting Long-Term Remission by Measuring Immediate Postoperative Growth Hormone Levels and Oral Glucose Tolerance Test in Acromegaly. Neurosurgery (2012) 70:1106–13; discussion 1113. doi: 10.1227/NEU.0b013e31823f5c16 22067418

[20] Cunha MLVD Borba LAB Boguszewski CL. Random Gh and Igf-I Levels After Transsphenoidal Surgery for Acromegaly: Relation With Long-Term Remission. Endocrine (2020) 68:182–91. doi: 10.1007/s12020-020-02227-2 32078118

[21] Feelders RA Bidlingmaier M Strasburger CJ Janssen JA Uitterlinden P Hofland LJ. Postoperative Evaluation of Patients With Acromegaly: Clinical Significance and Timing of Oral Glucose Tolerance Testing and Measurement of (Free) Insulin-Like Growth Factor I, Acid-Labile Subunit, and Growth Hormone-Binding Protein Levels. J Clin Endocrinol Metab (2005) 90:6480–9. doi: 10.1210/jc.2005-0901 16159936

[22] van Esdonk MJ van Zutphen EJM Roelfsema F Pereira AM van der Graaf PH Biermasz NR. How Are Growth Hormone and Insulin-Like Growth Factor-1 Reported as Markers for Drug Effectiveness in Clinical Acromegaly Research? A Comprehensive Methodologic Review. Pituitary (2018) 21:310–22. doi: 10.1007/s11102-018-0884-4 PMC5942341 29605877

[23] Cambria V Beccuti G Prencipe N Penner F Gasco V Gatti F. First But Not Second Postoperative Day Growth Hormone Assessments as Early Predictive Tests for Long-Term Acromegaly Persistence. J Endocrinol Invest (2021) 44:2427–33. doi: 10.1007/s40618-021-01553-0 PMC8502138 33837920

[24] Asha MJ Takami H Velasquez C Oswari S Almeida JP Zadeh G. Long-Term Outcomes of Transsphenoidal Surgery for Management of Growth Hormone-Secreting Adenomas: Single-Center Results. J Neurosurg (2019) 1–11. doi: 10.3171/2019.6.JNS191187 31604330

[25] Theodosopoulos PV Leach J Kerr RG Zimmer LA Denny AM Guthikonda B. Maximizing the Extent of Tumor Resection During Transsphenoidal Surgery for Pituitary Macroadenomas: Can Endoscopy Replace Intraoperative Magnetic Resonance Imaging? J Neurosurg (2010) 112:736–43. doi: 10.3171/2009.6.JNS08916 19835472

[26] Araujo-Castro M Pascual-Corrales E Martínez-Vaello V Baonza Saiz G Quiñones de Silva J Acitores Cancela A. Predictive Model of Surgical Remission in Acromegaly: Age, Presurgical GH Levels and Knosp Grade as the Best Predictors of Surgical Remission. J Endocrinol Invest (2021) 44:183–93. doi: 10.1007/s40618-020-01296-4 32441006

[27] Antunes X Ventura N Camilo GB Wildemberg LE Guasti A Pereira PJM. Predictors of Surgical Outcome and Early Criteria of Remission in Acromegaly. Endocrine (2018) 60:415–22. doi: 10.1007/s12020-018-1590-8 29626274

[28] Qiao N Shen M He W He M Zhang Z Ye H. Machine Learning in Predicting Early Remission in Patients After Surgical Treatment of Acromegaly: A Multicenter Study. Pituitary (2021) 24:53–61. doi: 10.1007/s11102-020-01086-4 33025547

[29] Yao S Chen WL Tavakol S Akter F Catalino MP Guo X. Predictors of Postoperative Biochemical Remission in Acromegaly. J Neurooncol (2021) 151:313–24. doi: 10.1007/s11060-020-03669-4 PMC10077515 33394265

[30] Agrawal N Ioachimescu AG. Prognostic Factors of Biochemical Remission After Transsphenoidal Surgery for Acromegaly: A Structured Review. Pituitary (2020) 23:582–94. doi: 10.1007/s11102-020-01063-x 32602066

